# Synchrotron X-Ray Visualisation of Ice Formation in Insects during Lethal and Non-Lethal Freezing

**DOI:** 10.1371/journal.pone.0008259

**Published:** 2009-12-14

**Authors:** Brent J. Sinclair, Allen G. Gibbs, Wah-Keat Lee, Arun Rajamohan, Stephen P. Roberts, John J. Socha

**Affiliations:** 1 Department of Biology, The University of Western Ontario, London, Ontario, Canada; 2 School of Life Sciences, University of Nevada-Las Vegas, Las Vegas, Nevada, United States of America; 3 X-Ray Science Division, Argonne National Laboratory, Argonne, Illinois, United States of America; 4 Department of Engineering Science and Mechanics, Virginia Polytechnic Institute and State University, Blacksburg, Virginia, United States of America; Universidad Europea de Madrid, Spain

## Abstract

Although the biochemical correlates of freeze tolerance in insects are becoming well-known, the process of ice formation *in vivo* is subject to speculation. We used synchrotron x-rays to directly visualise real-time ice formation at 3.3 Hz in intact insects. We observed freezing in diapausing 3^rd^ instar larvae of *Chymomyza amoena* (Diptera: Drosophilidae), which survive freezing if it occurs above −14°C, and non-diapausing 3^rd^ instar larvae of *C. amoena* and *Drosophila melanogaster* (Diptera: Drosophilidae), neither of which survive freezing. Freezing was readily observed in all larvae, and on one occasion the gut was seen to freeze separately from the haemocoel. There were no apparent qualitative differences in ice formation between freeze tolerant and non-freeze tolerant larvae. The time to complete freezing was positively related to temperature of nucleation (supercooling point, SCP), and SCP declined with decreasing body size, although this relationship was less strong in diapausing *C. amoena*. Nucleation generally occurred at a contact point with the thermocouple or chamber wall in non-diapausing larvae, but at random in diapausing larvae, suggesting that the latter have some control over ice nucleation. There were no apparent differences between freeze tolerant and non-freeze tolerant larvae in tracheal displacement or distension of the body during freezing, although there was markedly more distension in *D. melanogaster* than in *C. amoena* regardless of diapause state. We conclude that although control of ice nucleation appears to be important in freeze tolerant individuals, the physical ice formation process itself does not differ among larvae that can and cannot survive freezing. This suggests that a focus on cellular and biochemical mechanisms is appropriate and may reveal the primary adaptations allowing freeze tolerance in insects.

## Introduction

The ability of some insects to survive internal ice formation has been known since the 18^th^ century [1]. In spite of considerable advances in understanding the biochemical correlates to this freeze tolerance [2], there remain significant gaps in our understanding of the process of freezing and how freeze tolerance is achieved. A general model of freeze tolerance suggests that ice formation is restricted to extracellular spaces, resulting in osmotic dehydration of cells [3]. Carbohydrate cryoprotectants are thought to protect membranes and proteins during this dehydration as well as to change the aqueous properties of the cell [4]. Ice nucleators may allow the controlled initiation of freezing, antifreeze proteins prevent unfavourable growth and redistribution of ice as a result of recrystallisation [5], and phospholipid remodelling affects membrane phase and behaviour at low temperatures [6]. Recently, aquaporins have been implicated as key in freeze tolerance, perhaps by facilitating osmotic equilibration of cells during the freezing process [7], [8]. However, none of these cellular-level adaptations appear to be both necessary and sufficient for freeze tolerance, and it is unclear whether organismal-level ice formation processes are important in determining freezing survival.

Although there are many biochemical responses that may mitigate the effects of freezing on tissues and cells, ice formation is undeniably a physical process. Understanding the *in vivo* process of ice formation, and the extent to which these processes differ among insects that do and do not survive freezing, is therefore an important aspect of understanding the freezing process. There have been several light microscopy studies of the relationship between ice formation and insect cells. Sinclair & Wharton [9] showed that Malpighian tubule cells of *Hemideina maori* are dehydrated when frozen *in vivo*, whereas survivable intracellular ice formation has been demonstrated in tissues of cockroaches [10] and gall flies [11].

Several alternatives to light microscopy have been employed to visualise ice and freezing in frozen insects. X-ray spectroscopy has been used to examine freezing and lipid coalescence *in vivo* in *Drosophila triauraria*, confirming that ice formation kills these freeze avoiding insects, whereas fat crystallisation at higher temperatures does not [12]. Thermal imaging can be used to observe freezing in intact insects without physical contact [13]. Mietchen *et al*. [14] used *in vivo* magnetic resonance imaging (MRI) to observe the three-dimensional location of ice in frozen *Eurosta solidaginis* and *Epiblemma scudderiana*. However, each of these techniques has inherent limitations. X-ray spectroscopy integrates across the entire organism, as do nuclear magnetic resonance spectroscopy [15] and differential scanning calorimetry [16]. Thermal imaging [e.g. 13] allows a view only of the outside of the animal, often at relatively low resolution, while MRI lacks the temporal resolution to observe ice formation processes in real time [14]. To our knowledge, none of these techniques has allowed real-time observation of ice formation in intact insects.

Recent advances in synchrotron x-ray phase-contrast imaging have significantly improved the ability to observe internal processes in insects, including respiratory tracheal compression and internal fluid dynamics of digestion and circulation [17], [18]. Because the water-to-ice transition is a phase transition, it is readily observed using this technique, and thus provides an opportunity to observe ice formation processes in insects at high spatial and temporal resolutions. Despite limitations of the technique, in particular the inability to distinguish some soft structures within the insect's body [17], synchrotron x-ray phase-contrast imaging provides the first opportunity to observe ice formation processes directly and to compare these between insects that do and do not survive freezing.

In this study, we use 2D synchrotron x-ray phase-contrast imaging to directly observe internal ice formation in insect larvae. In particular, we compare diapausing larvae of the fruit fly *Chymomyza amoena* (Diptera, Drosophilidae), which survive internal ice formation [19], with non-diapausing *C. amoena* larvae and larvae of *Drosophila melanogaster*, neither of which survive freezing. We examine the rate of ice formation across temperatures, as well as the progression of the freezing process and the amount of displacement of internal structures and physical distension as a result of freezing. Our hypothesis was that there would be significant, detectable differences in physical ice formation processes between individuals that do and do not survive freezing.

## Results

Diapausing *C. amoena* larvae survived freezing under the conditions identical to those used in our synchrotron observations 85% of the time (n = 13, mean±s.e.m. SCP: -9.1±0.85°C), whereas non-diapausing larvae never survived freezing (n = 12, SCP: −14.5±1.51°C). None of the surviving diapausing larvae had SCPs below −14°C. Larvae did not survive exposure to the x-ray beam during the long duration of our experiments, but survived the cold exposure under identical conditions without the beam. Some of the individuals in the x-ray observations froze below -14°C (Table 1), so all statistical analyses were performed twice: using all data, and using data only from those individuals that froze at temperatures above −14°C.

**Table 1:** Supercooling points (SCP) of diapausing and non-diapausing *Chymomyza amoena* and *Drosophila melanogaster* larvae during synchrotron observations of freezing process.

	SCP (overall), °C				SCP (above −14°C), °C			
	N	Mean (±sem)	min	max	N	Mean (±sem)	min	max
**Diapausing ** ***C. amoena***	14	−10.4±1.8	−23.9	−2.4	10	−6.9±1.1	−11.3	−2.4
**Non-diapausing ** ***C. amoena***	12	−11.7±2.2	−21.4	−2.5	7	−6.1±1.5	−12.8	−2.5
***D. melanogaster***	9	−10.5±2.6	−23.3	−4.2	6	−5.7±0.9	−8.5	−4.2

### Qualitative Observations of Freezing

**Figure 1:**
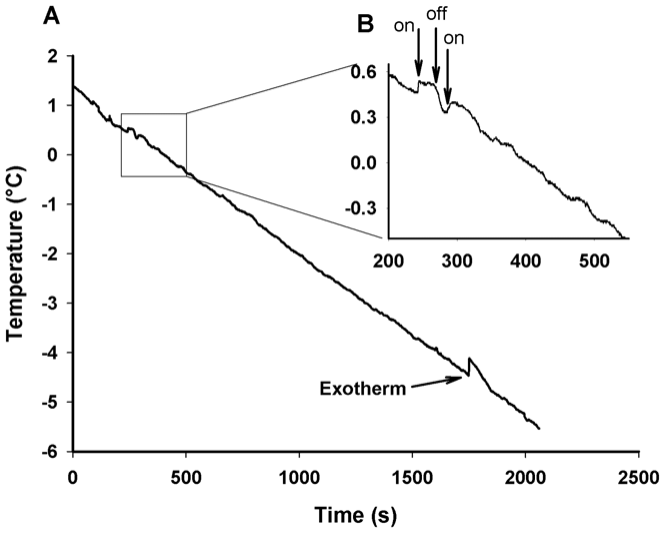
Thermal effect of synchrotron x-rays. Example of thermal effect of synchrotron x-ray beam (inset, beam being switched on and off marked with arrows) and exotherm detected from freezing of a *Chymomyza* larva. The SCP was the lowest temperature prior to the onset of freezing; this individual froze at −4.5°C.

Exposure to the x-ray beam resulted in a slight (c. 0.1°C) increase in temperature of the larvae (Figure 1). Preliminary experiments indicated that larvae survived brief exposures to the x-ray beam, but were killed by the prolonged exposures (>1 h) used in our study. The exact timing of mortality was not known; during a cooling trial, larvae entered chill coma and ceased moving, and we were thus unable to determine mortality due to beam exposure from our x-ray imaging alone.

**Figure 2:**
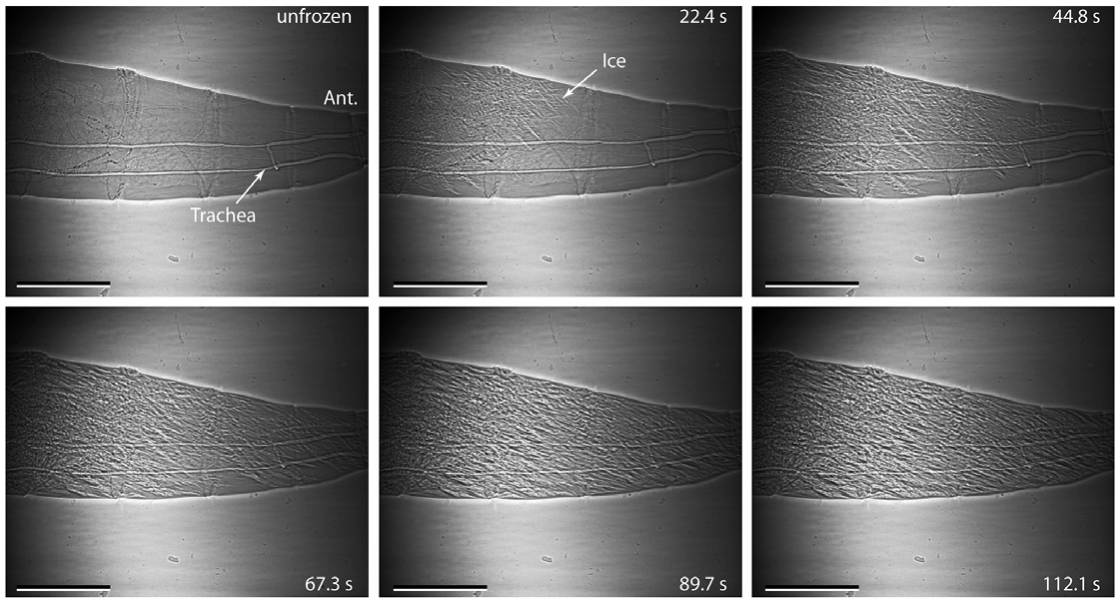
Spicular ice formation in *Chymomyza amoena*. Ice formation in a diapausing larva of *Chymomyza amoena*, showing formation of large, spicular ice crystals. This individual froze at -3.3°C. Time after freezing shown in seconds. ‘Ant.’ indicates the anterior end of the larva. The scale bar is 0.5 mm. The complete freezing sequence for this individual can be viewed in Movie S1.

**Figure 3:**
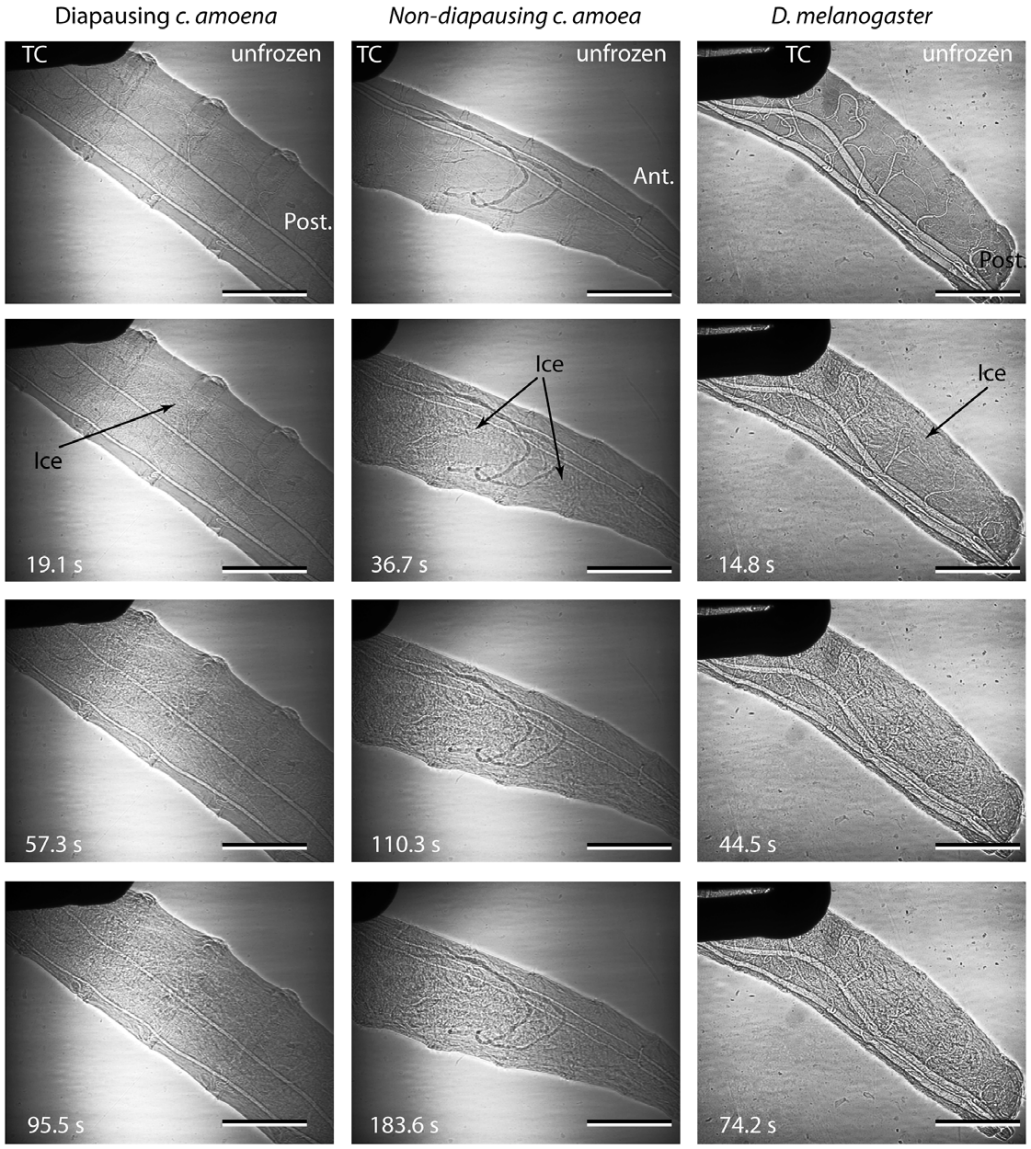
Ice formation at high sub-zero temperatures. Representative examples of ice formation processes at relatively high sub-zero temperatures in a diapausing larva of *C. amoena* (left column; SCP −2.4°C, also Movie S2), non-diapausing *C. amoena* larva (middle column; SCP −3.7°C, also Movie S3) and *D. melanogaster* (right hand column; SCP −4.2°C, also Movie S4). ‘Ant.’ and ‘Post.’ indicate anterior or posterior ends of the larvae; TC indicates the thermocouple, and the scale bar is 0.5 mm.

**Figure 4:**
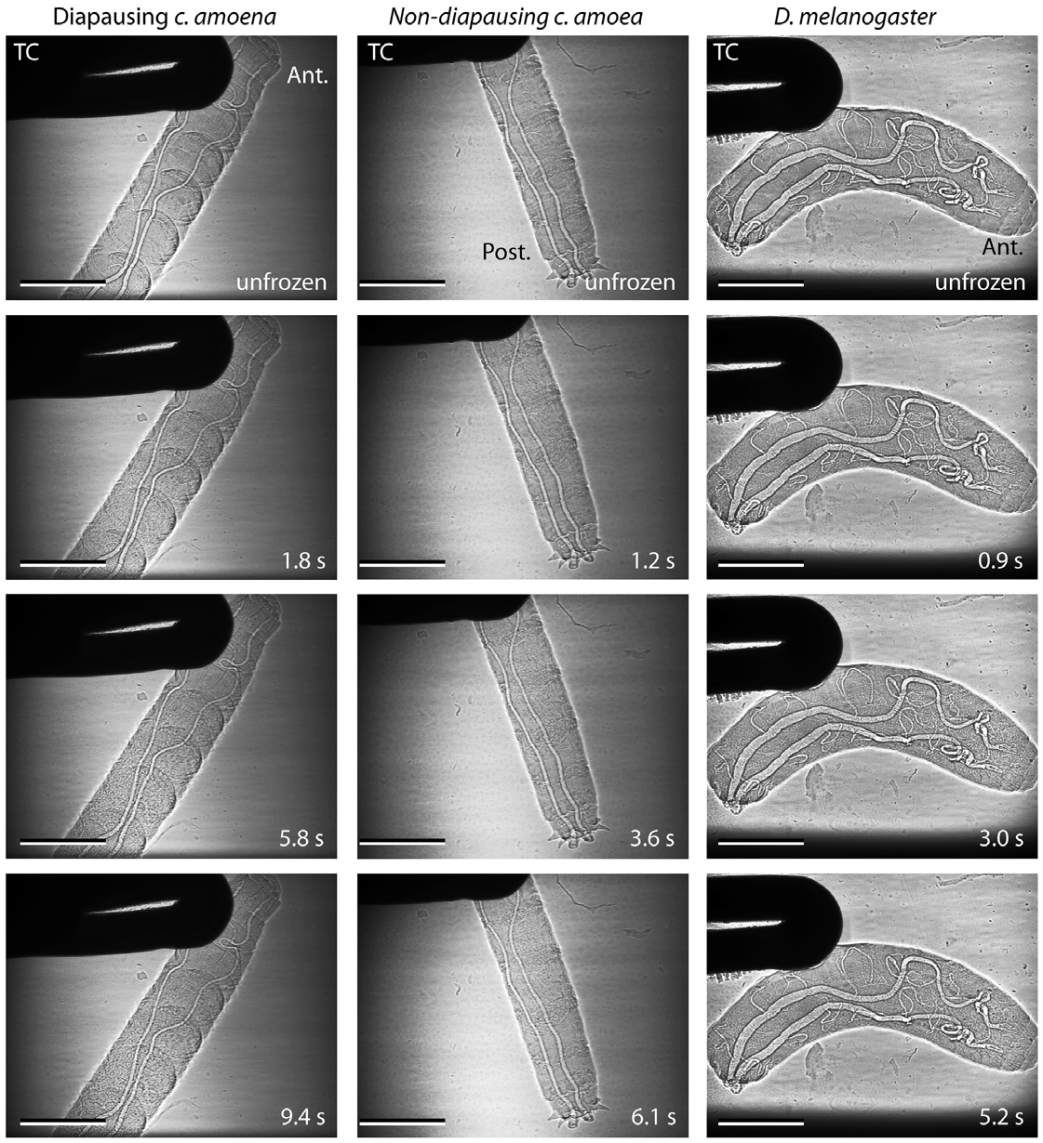
Ice formation at low sub-zero temperatures. Representative examples of ice formation processes at relatively low sub-zero temperatures in a diapausing larva of *C. amoena* (left column; SCP −16.5°C, also Movie S5), non-diapausing *C. amoena* larva (middle column; SCP −17.3°C, also Movie S6) and *D. melanogaster* (right hand column; SCP −21.6°C, also Movie S7). ‘Ant.’ and ‘Post.’ indicate anterior or posterior ends of the larvae; TC indicates the thermocouple and the scale bar is 0.5 mm. Note that the time to complete freezing was much shorter than in the individuals in Figure 3.

The air-filled tracheae of the larvae were clearly visible using phase-contrast x-ray imaging, and occasionally darker structures, which may be soft structures such as Malpighian tubules or developing salivary glands, were visible in all species. Ice formation was readily observed in the freezing larvae as a change in the appearance of the image that coincided with the onset of the exotherm. Sometimes the change was simply a change in the larva to a more granular appearance, but more often ice crystals could be discerned (Figures 2, 3, 4). At the onset of freezing, the larva often shifted position, possibly as a result of ice formation at the larva-thermocouple interface, and straightened, probably in response to expansion of internal water with freezing.

The quality of ice varied among trials, with two individuals of *C. amoena* (one diapausing, one non-diapausing) that froze at relatively high subzero temperatures displaying distinctive spicular-shaped patterns (e.g. Figure 2, Movie S1). Because the x-ray image is a two-dimensional projection of the three-dimensional insect, it was not possible to make accurate measurements of ice crystal size, nor was it possible to accurately measure the rate of spread of ice fronts due to overlap of ice in the (indistinguishable) third dimension.

**Figure 5:**
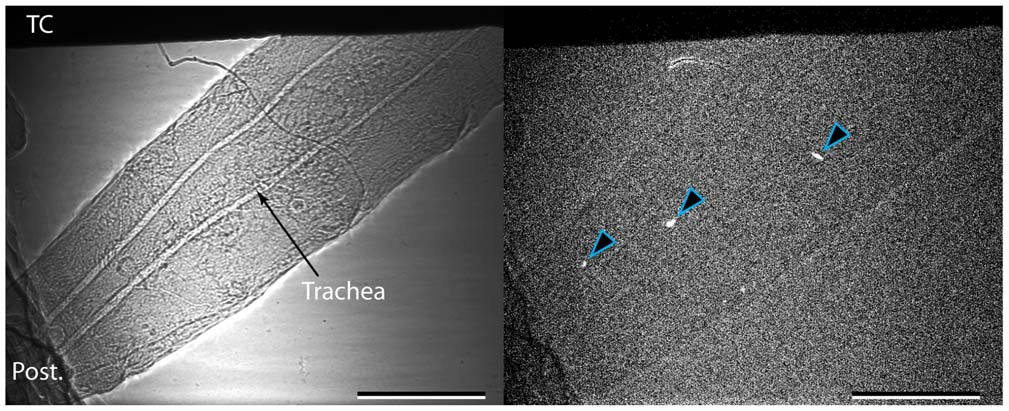
Isolated freezing events during freezing. Example of isolated freezing events visible in some ice formation sequences, in this case in a diapausing *C. amoena* larva with an SCP of −8.1°C. Left hand image is the phase-contrast x-ray image, right hand is a subtraction of two consecutive images collected c. 0.3 s apart, and white patches (indicated with arrows) are freezing events that appear to be an entire compartment of water freezing. These events are also visible in movies S1, S2, S3 and S4. TC indicates the thermocouple, ‘Post.’ the posterior end, which in this case is in contact with the wall of the chilling chamber. Scale bar is 0.5 mm.

**Figure 6:**
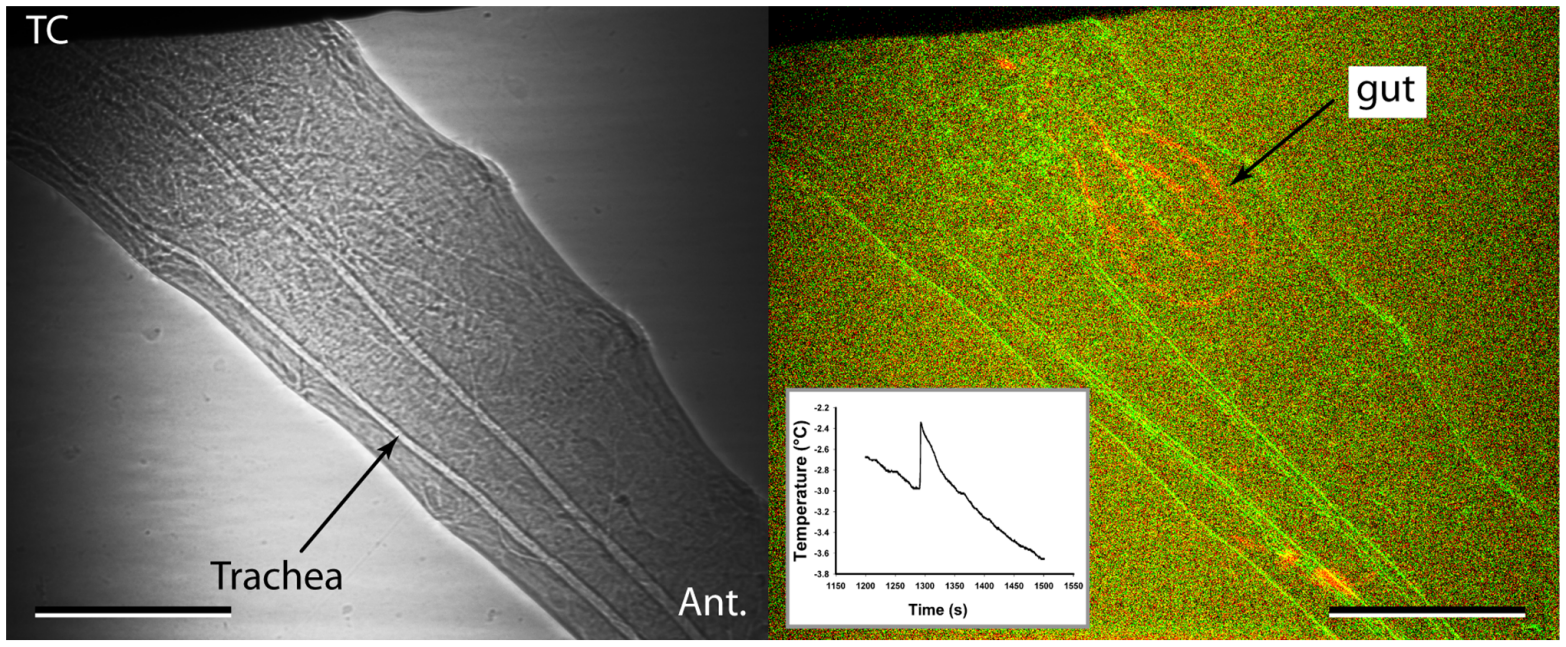
Ice formation in gut during freezing. Apparent gut freezing in a non-diapausing *C. amoena* larva. Left hand image is the x-ray image, right hand is a composite of subtraction images of the frames where the gut appeared to freeze (orange) and the initial freezing frame (green), which shows the locations of the cuticle and tracheal landmarks. The individual froze at −3.0°C, and the gut froze 67.6 s after the initiation of freezing; inset shows exotherm from this individual, note the lack of a double exotherm. ‘Ant.’ indicates the anterior of the animal, TC indicates the thermocouple.

In non-diapausing *C. amoena* (10 of 12 cases) and *D. melanogaster* (all cases, n = 9), ice formation appeared to begin at the thermocouple or a point of contact with the chamber wall regardless of the SCP. However, in diapausing *C. amoena*, nucleation was at a point of contact in only half of cases (7 of 14 cases). There did not appear to be a consistent point of nucleation in the other seven individuals, although in several cases the nucleation point was out of the field of view. The proportion of individuals where freezing was initiated at a contact point was significantly different from that in the non-freeze tolerant individuals (χ^2^ = 7.9, df = 2, p = 0.019). SCP did not differ significantly in diapausing *C. amoena* between individuals where freezing was initiated at contact points (−9.2°C±2.7) and those where it was not (−11.5°C±2.4; t_12_ = 0.634, p = 0.538). At the lowest SCPs, it was sometimes difficult to discern the progression of freezing because of small crystals and rapid freezing (e.g. Figure 4). In cases where ice propagation was visible, freezing appeared to proceed outwards from the point of nucleation, but ice formation continued throughout the animal during the ice formation process. This was often in the form of isolated units, which were often large (100 µm or greater, indicating larger populations of water) but there were also a number of smaller events with largest diameters of 27.2±0.9 µm (mean and standard error of 43 events from 8 diapausing and non-diapausing *C. amoena*; Figure 5). On one occasion (Figure 6), freezing of a non-diapausing *C. amoena* appeared to occur separately in the gut and the haemocoel, but this was the only occasion on which we were able to discern any distinct compartmentalised freezing that might indicate differential freezing in soft tissues. There were no obvious qualitative differences in nucleation or ice formation between diapausing and non-diapausing *C. amoena*, or between *C. amoena* and *D. melanogaster*.

### Body Size, SCP and Time to Complete Freezing

**Figure 7:**
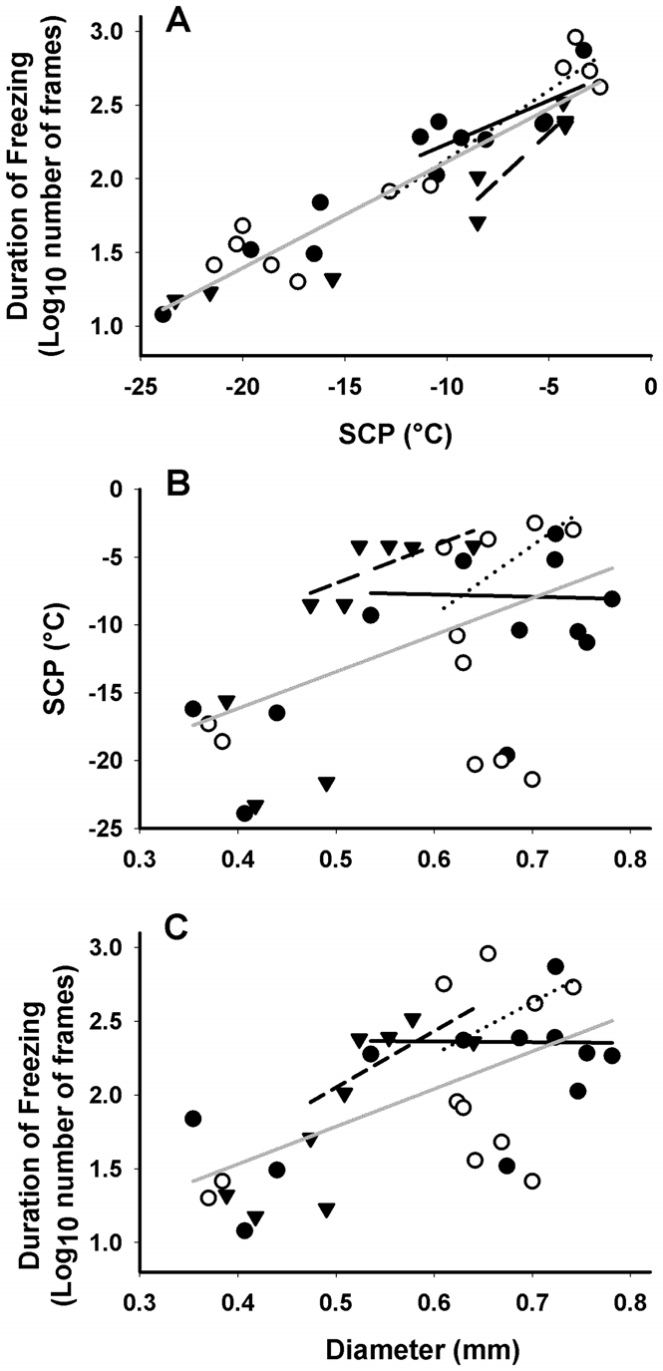
Duration of freezing depends on freezing temperature. Relationship between supercooling point (SCP), body size (as larval diameter) and duration of freezing events (number of frames; capture rate was approximately 3.3 Hz) in diapausing (filled circles, solid line) and non-diapausing (open circles, dotted line) *Chymomyza amoena* and *Drosophila melanogaster* (triangles, dashed line) larvae. Short lines indicate relationships for SCP >−14°C, grey line indicates overall relationship for the combined dataset. Overall analysis was performed as a GLM, and both body size (F_1,20_ = 13.82, p = 0.0014) and SCP (F_1,20_ = 267, p<0.001) were significant predictors of duration of freezing for the whole dataset and for SCP >−14°C (Body size: F_1,8_ = 11.6, p = 0.009; SCP: F_1,8_ = 53.87, p<0.0001). Above −14°C, there was no significant relationship between body size and SCP (F1,17 = 3.89, p = 0.065), and the slopes did not differ significantly among states (F_2,17_ = 0.89, p = 0.430).

Larger larvae took longer to freeze, and freezing took longer at higher temperatures than at lower temperatures (Figure 7). When body size and SCP are taken into account there was no significant difference in time taken to freeze among diapausing, non-diapausing *C. amoena* and *D. melanogaster* (F_2,20_ = 2.50, p = 0.108). This relationship becomes near-significant if low SCPs are excluded from the analysis (F_2,8_ = 3.49, p = 0.081) and *post hoc* analysis (p<0.1) suggests that the difference was among species rather than being attributable to the capacity to survive freezing. Importantly, at SCPs above −14°C, there was no relationship between body size and SCP in diapausing *C. amoena* larvae, which differentiates the freeze tolerant larvae from those that cannot survive freezing (Figure 7).

### Distension and Displacement as a Result of Freezing

**Figure 8:**
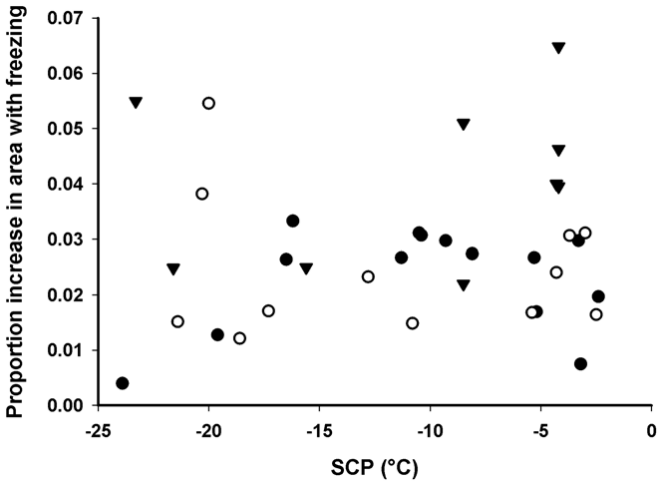
Freezing temperature does not determine distension. Effect of supercooling point (SCP) on distension with freezing in insect larvae that do and do not survive freezing. There was no relationship between SCP and the proportional increase in area (used as a proxy for whole-body distension) with freezing in diapausing (filled circles) or non-diapausing (open circles) *C. amoena*, or in *D. melanogaster* larvae (triangles).

**Figure 9:**
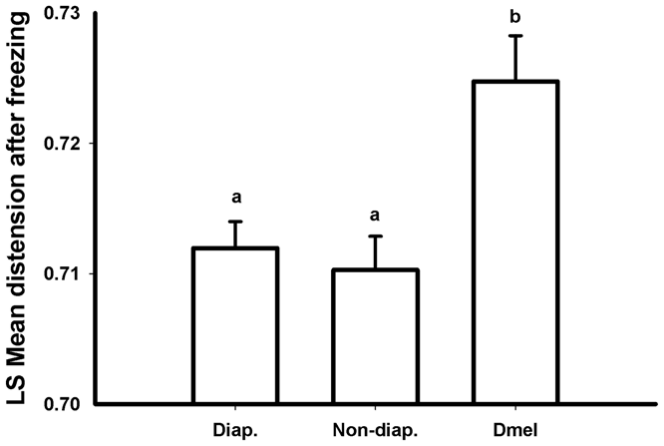
Distension with freezing in *D. melanogaster* and *C. amoena*. Comparison of distension resulting from freezing in insect larvae that do and do not survive freezing. Distension as a result of freezing was greater in *D. melanogaster* (Dmel) than in either diapausing (Diap.) or non-diapausing (Non-diap.) *Chymomyza amoena*. Least squares means are from an ANCOVA with initial area as a covariate; different letters indicate statistically significant differences. Only larvae that froze above −14°C are included.

In all of the runs, a noticeable lateral distension of the larva was visible as it froze (see Movies S1, S2, S3, S4, S5, S6, and S7; this is also visible in the subtraction images as a ‘flash’ of the tracheae). This lateral distension was quantified as a change in the sectional area within cuticular landmarks, and was not related to SCP (Figure 8, full dataset: F_1,27_ = 0.22, p = 0.644; >−14°C: F_1,15_ = 0.13, p = 0.722). However, there was significantly greater lateral distension in *D. melanogaster* than in diapausing or non-diapausing *C. amoena*, which did not differ (Figure 9; full dataset: F_2,27_ = 4.33, p = 0.023; >−14°C: F_2,15_ = 5.17, p = 0.020; Tukey's *post hoc* test p<0.05). Because of the restricted field of view, we were unable to estimate longitudinal distension, which presumably accounts for the interspecific difference in lateral distension with ice formation.

Displacement of visible internal structures relative to one another and to cuticular landmarks was apparent for all states/species. The overall movement of the larva, including distension, was accounted for prior to analysis of displacement. Relative displacement was unrelated to body size, state (species and diapause status) or SCP (ANCOVA: p>0.08 in all cases), both with the whole dataset and with SCPs above −14°C. 

## Discussion

Here we provide the first real-time images of internal ice formation in intact insects. The quality of ice was variable, ranging from large spicular crystals to more subtle (presumably smaller) crystals, and although this variation was to some extent dependent upon nucleation temperature, it did not appear to be related to the ability to tolerate internal ice formation. The relationship between SCP and freezing duration and the extent of displacement of visible internal structures also did not differ among the different species and states. Lateral distension of larvae upon freezing differed significantly between *D. melanogaster* and *C. amoena*, but did not differ among diapausing and non-diapausing larvae of the latter, suggesting that it does not relate to freezing tolerance. However, diapausing larvae of *C. amoena* differed from non-diapausing and *D. melanogaster* in their ice nucleation characteristics. First, ice formation did not appear to be initiated at points of contact in diapausing *C. amoena*, whereas it was in non-diapausing and *D. melanogaster*. Second, there was no significant relationship between body size and SCP for diapausing *C. amoena* above −14°C, where they would expect to survive freezing, whereas there was a stronger (but also non-significant, see Figure 7) relationship in non-diapausing and *D. melanogaster*. Thus, control of ice nucleation appears to be the sole organismal-level attribute we can measure that is present only in freeze-tolerant individuals. A number of authors [e.g. 3], [20] have hypothesised that the primary difference among cold tolerance strategies in insects lies in ice nucleation. Here we provide evidence that control of nucleation appears to be a component of freezing survival within a species.

Freeze tolerance is generally associated with high SCPs, and in *C. costata* requires external inoculation for survival [21], so the low SCPs we observed in diapausing individuals are surprising. Since these larvae overwinter in a moist habitat [19], inoculative freezing is likely, although the decoupling of body size and SCP in diapausing *C. amoena* (Figure 7) suggests the presence of endogenous ice nucleators. Nevertheless, we repeated all analyses comparing only individuals that froze above −14°C, since experiments outside the beam did not yield SCPs below this value in *C. amoena*. In non-diapausing *C. amoena* and *D. melanogaster* larvae, nucleation generally began at the thermocouple or contact point with the chamber wall, even in individuals that froze at low temperatures. This is likely because heat is conducted more efficiently at these points than to the surrounding air, and the broad range of temperatures do not indicate inoculative freezing, which tends to happen consistently at high subzero temperatures when it occurs [22]. However, if nucleation is initiated by the contact at temperatures that differ from the non-contact SCP, then this would have ramifications for all studies of cold tolerance that measure SCPs, as the contact between the animal and its surroundings would need to mimic natural environments, and would not be easily solved by using alternative methods of detecting ice formation like thermal imaging or differential scanning calorimetry [13], [23].

Although 2D synchrotron x-ray phase-contrast imaging does not allow us to distinguish all soft-tissue structures without the aid of contrast agents, the gut was observed to freeze on at least one occasion (Figure 6). Multiple exotherms are often observed in large insects (B.J. Sinclair, unpublished observations), and in plants are ascribed to the freezing of isolated populations of water molecules [24]. However, in the case presented in Figure 6, no concomitant second exotherm was detected in association with the gut freezing. This may be because other freezing processes were still in progress, obscuring detection of subsidiary ice formation events, or because the amount of water confined to the gut is too small to be detectable in such small larvae. Nevertheless, the observable freezing of the gut was not a common event, suggesting that there is no consistent barrier to ice propagation between the haemocoel and gut in these larvae. In many cases (particularly slow freezing events at high subzero temperatures), numerous isolated freezing events were observed (Figure 5). We interpret these as the freezing of small compartments of fluid, although their nature is uncertain. The events are not confined to any one part of the body, and they appear to be too large to be individual cells, except possibly those of the salivary gland or fat body.

We observed significant distension of larvae with ice formation, and some displacement of visible internal structures (i.e. tracheae). This distension and displacement likely reflect the significant physical perturbations inside an insect during ice formation, and distension was more marked in *D. melanogaster* than *C. amoena*, regardless of state. Thus, some degree of control of ice formation may be associated with the ability to survive ice formation, although this may also reflect differences in the propensities of the two species to expand longitudinally during freezing. In particular, the tracheae were not partially or fully collapsed by the expansion of water upon freezing, which could allow gas exchange in the frozen state, and also suggests that the burst of CO_2_ seen in caterpillars when freezing [25] is not necessarily a result of a mechanically-induced expulsion of air from the tracheal system.

The strength of the relationship between body size and SCP indicates that a physical attribute (body size) is a primary determinant of freezing in non-diapausing *C. amoena* and *D. melanogaster*, as has been shown previously for the relationship between body size, water content and SCP [3], [26], [27]. However, the body size – SCP relationship appears to break down in SCPs above −14°C, particularly in diapausing *C. amoena*, which suggests an independent control on nucleation temperature, such as ice nucleation agents [5]. Preliminary investigations indicate the presence of an endogenous ice nucleator in diapausing *C. costata* (A. Rajamohan & B.J. Sinclair, unpublished) and *C. amoena* larvae (C. Warshafsky & B.J. Sinclair, unpublished). Control of nucleation and initiation of freezing at a high temperature has often been suggested as a key component of freeze tolerance [see 3], [28], and our results support the notion that higher nucleation temperatures result in a slower freezing process, and that individuals that survive freezing at least partly manipulate nucleation. We do note that the lack of relationship between body size and duration of ice formation in diapausing *C. amoena* above −14°C appears to imply that the rate of ice formation is faster in larger larvae. However, the duration of freezing encompasses the period from the start of ice formation to the last isolated freezing event. Because the isolated freezing events occur sporadically over a long period of time, particularly at high SCPs (see, e.g., Movies S1 and S2), the rate of bulk ice formation may be obscured in these individuals.

Aside from apparent manipulation of nucleation in diapausing *C. amoena*, there were no obvious differences in ice formation between diapausing and non-diapausing *C. amoena* larvae that can account for the ability of the former to survive internal ice formation. This implies that the major differences allowing ice formation in diapausing individuals are either manifest in the interaction between the forming ice and the soft tissues and organs (invisible to this technique), or that the key adaptations to freeze tolerance occur at the cellular physical, biochemical or molecular level. There were some differences in distension between *C. amoena* and *D. melanogaster*, but until further species are examined, it is unclear whether this represents a significant difference in the localisation and control of freezing, or just interspecific variation in the ability of the body to expand with ice formation.

Phase-contrast synchrotron x-ray imaging is able to provide detailed, real-time images of ice formation in intact insects. The addition of contrast agents [see 17,29], and recordings at higher temporal or spatial resolution will allow further development of this technique, and may allow the resolution of soft tissues and cells during the freezing process. The long depth of focus of the image makes it difficult to resolve spatial patterns of ice formation in the plane parallel to the x-rays, and hampered our attempts to measure the rate of spread of the ice front. However, a use of this technique in combination with other other imaging techniques that operate at different sensitivities and spatio-temporal resolutions, for example MRI [14], x-ray spectroscopy [12] and X-ray tomography [30], may allow a more thorough reconstruction of the three-dimensional process of freezing.

Although there is negligible heat build-up associated with beam exposure, exposure to high intensity x-rays causes damage to biomolecules and, ultimately, the death of the organism. Preliminary experiments indicated that larvae survive brief (10–20 min) exposures under the experimental conditions, but survival for the duration of the experiment is unlikely, and the cause of mortality is unknown. During our experiments, the larvae entered chill coma early in the cooling process, so it is not possible to pinpoint time to mortality from our recordings. Nevertheless, our data provide insight into the physical aspects of freezing. Assuming that the x-ray beam allows cell membranes and ion channels to remain intact and functional for the duration of the experiment, then our observations should be reflective of any passive processes of ice formation, including flow of ions and water into and out of cells and the function of any ice-active proteins (bearing in mind the likely very low activation energies). Responses to ice formation appear to be active in vertebrates and earthworms, with ice formation resulting in changes in gene expression and carbohydrate metabolism [e.g. 15], [31], [32]. Freezing processes in insects are thought to be largely passive [3], so our observations on intact larvae that may have died during imaging are likely reflective of the behaviour of ice in the living organism.

Finally, like many other studies on insect cold tolerance, our focus was on freezing processes. However, much damage in mammalian and cryopreserved cells occurs during reperfusion/thawing [33], and it is possible that thawing is also a key aspect of survival of the freezing process in insects. Because of the long duration of melting compared to freezing, the ice formation process is more easily discerned than the thawing process using the present techniques, but the latter still involves a change in phase of the water, and investigation should be feasible.

### Conclusions

This study is the first to use phase-contrast synchrotron x-ray techniques to visualize ice formation in intact insects that can survive freezing. There is high variability in the freezing process, but we have confirmed the basic relationship between ice formation and body size, and between SCP and ice formation processes. Furthermore, individuals of the freeze tolerant species show reduced distension during freezing compared to *D. melanogaster*, and the individuals that survive freezing appear to have some control over ice nucleation. Nevertheless, apart from some apparent control over ice nucleation, there are few striking differences among species that do and do not survive freezing during ice formation, which suggests that key adaptations during the freezing process lie at the cellular, molecular and biochemical levels.

## Materials and Methods

### Study Animals and Freezing Survival

*Chymomyza amoena* were obtained from the Tucson *Drosophila* stock center (now the *Drosophila* species stock center at the University of Califonia, San Diego), and were reared at 18°C, c. 50% RH, 16L∶8D on Wheeler-Clayton medium [34] until the second instar, when they were transferred to a cornmeal-sucrose-agar diet (1.5% Agar, 4% active yeast, 7.2% cornmeal, 6.4% sucrose, 0.1% sodium benzoate w/v, 0.2% propionic acid v/v). Diapause was induced in 3^rd^ instar individuals by transferring 2^nd^ instar larvae to 5°C and 24 h darkness for 45 days. Diapausing individuals, which usually appeared immobile and pale, were handled on ice before experiments. Non-diapausing 3^rd^ instar individuals were kept at 18°C, c. 50% RH and were actively feeding along the sides of the food vials. The wild-type Berlin K strain of *D. melanogaster* was obtained from the Bloomington *Drosophila* stock center, and reared at 21°C, c. 50% RH and 12L∶12D photoperiod. Feeding 3^rd^ instar larvae were used in experiments. All larvae were removed from the medium, rinsed in distilled water, and blotted dry before use in experiments. Diapausing *C. amoena* were handled on ice, while non-diapausing *C. amoena* and *D. melanogaster* were handled at room temperature.

*D. melanogaster* larvae do not survive internal ice formation [35], and 90% of Berlin K 2^nd^ and 3^rd^ instar larvae are killed by 2 h exposure to −7°C irrespective of ice formation [36]. Low temperature survival of *C. amoena* was first measured by cooling larvae under identical conditions to those used later for x-ray exposure experiments (see below). Survival was determined by successful development and eclosion as adults after larvae were cooled to −30°C at 0.5°C/min, removed to room temperature and placed individually on rearing medium and allowed to develop at 17°C, c. 50% RH, 17L∶7D photoperiod.

### Synchrotron X-Ray Imaging

Larvae were suspended from a 40 awg type-T thermocouple in a custom-built air-filled brass 10×10×5 mm chamber with an argon-filled double window of Kapton polyimide (DuPont) to allow x-ray transmission. Dry air was blown across the outer wall to reduce condensation. The brass chamber was cooled by methanol circulated from a Lauda RP3530 refrigerated circulator. As far as possible, the larva was only in contact with the thermocouple, although on some occasions the anterior or posterior of the larva was also in contact with the floor or wall of the chamber. The chamber was cooled from 0°C to −30°C at 0.5°C min^−1^ and freezing of the larva detected via the thermocouple as the release of the latent heat of crystallisation (the exotherm). The lowest temperature before the initiation of freezing was the supercooling point (SCP). The chamber and larva were placed on a translation stage in the path of the x-ray beamline 32-ID at the Advanced Photon Source, Argonne National Laboratory in Argonne, IL, USA. Phase-contrast x-ray imaging was performed using 15 keV x-rays from a Si(111) monochromator. The sample-detector distance was about 1 m. X-rays were converted to visible light using a 100 mm thick cerium-doped yttrium aluminum garnet single crystal, and imaged onto a CCD camera (Sensicam, Cooke Corp) using a 5× Mitutoyo objective and tube lens.

The x-ray energy was chosen to optimize image clarity, without regard to animal survivorship. From rough calculations based on x-ray energy and larval body size, we estimate that the incident power density was approximately 1 mW/mm^2^, providing a radiation dose rate of approximately 128 Gy/s. This is a much larger rate than that used in previous live-imaging studies, which aimed to optimise survival. Here, our aim was to record physical processes at fine resolution, so we opted for a beam strength that likely resulted in death of the larvae within 10-20 min of exposure. Images were captured at 3.3 Hz as sequences of 14-bit TIFF images and then downsampled to 8-bit images for analysis.

### Data Analysis

Images were analyzed using ImageJ software [37] and measurements calibrated to images of a 400-mesh grid, and supercooling points of larvae were determined from thermocouple traces. To identify small changes in the larvae that occurred from frame to frame, sequences of subtraction images were created using the StackDifference plugin in ImageJ. These subtraction images isolated pixels that had changed from the previous frame, allowing easy visualisation of changes associated with freezing events. These images also allowed us to determine the duration of freezing, by identifying the first and last frames in which freezing events occurred. The body size of each larva was estimated by measuring the widest diameter of the larvae that was visible. Whenever possible, qualitative estimates of the location of ice nucleation and other observations were recorded. In several cases, a small ice formation event was visible in one of the final three frames recorded. In these cases, the data were omitted from analyses of the duration of freezing, but the final frame was still utilised as an endpoint for measurements of distension and displacement. Distension in response to freezing was determined by measuring the area of the same portion of the larva in the frames immediately before the initiation of freezing and after the completion of the freezing event. Because the larva moved and distended during freezing, cuticle landmarks were used to ensure that the same segments were included in both measurements.

Larval movement and displacement of internal structures was quantified by analyzing a 1-px wide segment of each image using the MultipleKymograph plugin in ImageJ. A histogram of the greyscale values across this segment was extracted from six frames in a freezing sequence: the frame before the initiation of ice formation, the frame after the completion of ice formation, and at four equidistant points in between. The edges of larval features were detected as peaks in this histogram, and these peaks were used to calculate distance between features. The overall movement of the larva was estimated using the displacement of the left-hand edge of the larva. The peak positions were standardised to the edges of the larva (removing any effect of distension), and absolute displacement of each peak for each interval calculated.

All statistical analyses were performed in SAS (v. 9.1, SAS Institute Inc., Cary, NC, USA). The relationship between SCP and (log10-transformed) duration of the freezing event was compared among ‘states’ (diapausing or non-diapausing *C. amoena*, and *D. melanogaster*) using a general linear model with body size (diameter) as a covariate using PROC GLM. Distension (increase in cross-sectional area, log10 transformed) was compared among states using PROC GLM with SCP as a covariate. Displacement of internal structures and was compared among states and across time using a repeated-measures analysis of covariance (PROC GLM) with body size as a covariate and SCP and state as factors in the model.

## Supporting Information

Movie S1 Spicular ice formation in a diapausing *Chymomyza amoena* larva. This individual froze at -3.3°C and is also shown in Figure 2. The left-hand frame is the x-ray image, the right-hand frame is a subtraction image showing (in white) differences between one frame and the next, which are attributable to ice formation events. The movie is at 9 frames per second, approximately 3× normal speed.
(9.90 MB MOV)

Movie S2 Freezing in a diapausing *C. amoena* larva, initiated at −2.4°C, also shown in the left-hand column of Figure 3. The left-hand frame is the x-ray image, the right-hand frame is a subtraction image showing (in white) differences between one frame and the next, which are attributable to ice formation events. The movie is at 9 frames per second, approximately 3× normal speed.
(9.73 MB MOV)

Movie S3 Freezing in a non-diapausing *C. amoena* larva, initiated at −3.7°C, also shown in the middle column of Figure 3. The left-hand frame is the x-ray image, the right-hand frame is a subtraction image showing (in white) differences between one frame and the next, which are attributable to ice formation events. The movie is at 9 frames per second, approximately 3× normal speed.
(9.77 MB MOV)

Movie S4 Freezing in a *Drosophila melanogaster* larva, initiated at −4.2°C, also shown in the right-hand column of Figure 3. The left-hand frame is the x-ray image, the right-hand frame is a subtraction image showing (in white) differences between one frame and the next, which are attributable to ice formation events. The movie is at 9 frames per second, approximately 3× normal speed.
(9.86 MB MOV)

Movie S5 Freezing in a diapausing *C. amoena* larva, initiated at −16.5°C, also shown in the left-hand column of Figure 4. The left-hand frame is the x-ray image, the right-hand frame is a subtraction image showing (in white) differences between one frame and the next, which are attributable to ice formation events. The movie is at 9 frames per second, approximately 3× normal speed.
(9.11 MB MOV)

Movie S6 Freezing in a non-diapausing *C. amoena* larva, initiated at -17.3°C, also shown in the middle column of Figure 4. The left-hand frame is the x-ray image, the right-hand frame is a subtraction image showing (in white) differences between one frame and the next, which are attributable to ice formation events. The movie is at 9 frames per second, approximately 3× normal speed.
(7.21 MB MOV)

Movie S7 Freezing in a *Drosophila melanogaster* larva, initiated at −21.6°C, also shown in the right-hand column of Figure 4. The left-hand frame is the x-ray image, the right-hand frame is a subtraction image showing (in white) differences between one frame and the next, which are attributable to ice formation events. The movie is at 9 frames per second, approximately 3× normal speed.
(5.45 MB MOV)
